# First *in-situ* observation of a moving natural pyroclastic density current using Doppler radar

**DOI:** 10.1038/s41598-019-43620-w

**Published:** 2019-05-14

**Authors:** Lea Scharff, Matthias Hort, Nick R. Varley

**Affiliations:** 1 CEN, Institut für Geophysik, Universität Hamburg, Hamburg, 20146 Germany; 2 CIIV, Facultad de Ciencias, Universidad de Colima, Colima, 28045 Mexico

**Keywords:** Natural hazards, Volcanology

## Abstract

Pyroclastic density currents are one of the most devastating volcanic hazards. Understanding their dynamics is a key to develop successful hazard mitigation strategies. The hazard associated with pyroclastic density currents is commonly investigated a posteriori from their deposits or a priori using analogue and numerical experiments. Despite the low probability of observing a natural moving pyroclastic density current, we present the first *in-situ* analysis of the internal particle velocities of pyroclastic density currents at Volcán de Colima using a Doppler radar. Our data show two Vulcanian explosions, immediately followed by column collapse and a first pyroclastic density current travelling down the south flank with an average speed of 30 m/s (>50 m/s maximum speed) to a distance of 3 km from the crater rim. The direction of the pyroclastic density current coincided with that of the radar beam enabling measurement of velocity spectra (histogram of particle velocities within the radar beam). The measurement geometry enables the simultaneous measurement of the dense undercurrent at the crater rim (with <20 m/s and an increasing echo power over 20 s) and the dilute cloud higher above the topography approaching the radar (with >20 m/s and approximately constant echo power). The presented data set may be used as a benchmark for future experimental and numerical models that simulate the dynamics of pyroclastic density currents. Using the measured velocities of the collapsing column as input for numerical models will permit the validation of the models for the prediction of the true run-out distance, and thus provide valuable information for hazard assessments.

## Introduction

Numerical models and analogue experiments show that both pyroclastic density currents (PDCs) and snow avalanches are comprised of a dense undercurrent, behaving like a granular flow, overlain by a dilute ash (or snow) cloud controlled by turbulence and entrainment. Besides the general similarities, three fundamental mechanisms for the generation of PDCs can be identified^1,2^: gravitational break-up of lava domes (so-called Merapi-type^3^), directed lateral explosions (e.g. the eruption of Mt. St. Helens, 18 May 1980^4,5^), and the gravitational collapse of eruption columns (e.g. during the Plinian eruption of Mt. Vesuvius, Italy, 79AD, which destroyed Pompeii^6^). During Merapi-type block-and-ash flows, large blocks break off the lava dome and fragment during their descent down the flank, thereby releasing volcanic gases^7^. In the case of a directed lateral explosion, an unstable, over-pressured lava dome collapses and releases volcanic gases and volcanic material explosively onto the flank^8^. The third type, the gravitational collapse of an eruption column, is a consequence of insufficient entrainment of ambient air into an initially non-buoyant, momentum-driven eruption column^1^.

The impact of PDCs on the local population and infrastructure^9^ and why they occur^10,11^ has been the focus of many research studies during the last decades. Up to now, the dynamics of PDCs have either been inferred from their deposits^11,12^ or were investigated using numerical models^13–18^ and small- to large-scale analogue experiments^19–23^. Experiments are typically equipped with video cameras for the analysis of dynamics, which is greatly hampered by the PDC’s opaqueness. To look into a PDC, remote sensing techniques such as Doppler radar can be used, however, they would require a much larger experimental setup, which is currently not possible for the volcanic case. So far only one comparable Doppler radar measurement exists: it reveals the internal dynamics of a snow avalanche (a controlled experiment) travelling down the flank of a fjord at 120 km/h^24^ (=33 m/s). This Doppler radar data set has subsequently been used to validate a numerical model for snow avalanches^25^, which is still used for hazard assessment^26^.

Similar to avalanches, a PDC’s dense undercurrent is largely controlled by the local topography (see Supplementary Fig. S1). The dilute cloud above it, however, can decouple from the dense flow and overtop topographic barriers. Using numerical modelling, it is possible to produce maps of areas potentially affected by PDCs^20^. Usually these maps are also used by scientists to find safe places, outside the possible pathways of PDCs to install monitoring equipment. For a profiling stationary instrument, like the Doppler radar, this means that the probability to observe one of these PDCs is very low. Until now, nobody has captured a collapsing eruption column, culminating in a PDC approaching a radar installation, thereby enabling the measurement of particle velocities during the collapse and within the PDC.

Here we present the first *in-situ* measured velocities of a PDC that occurred on 21 November 2014, 18:24 UTC, at Volcán de Colima, Mexico, during a Vulcanian explosion. The associated eruption column ascended to 7 km above the crater^27^ (10–11 km above sea level). The PDC descended down the south flank and split into the Montegrande and San Antonio ravines, reaching a run-out distance of 2.9 and 3.1 km, respectively^27,28^. The Doppler radar recorded almost continuously from March 2014 until it was destroyed in July 2015 by another sequence of PDCs^29–32^. The Doppler radar data of the 2014 event, presented and analysed here, is unique with respect to the recorded dynamics (e.g. temporal evolution of velocities). We identified several characteristic dynamic phases during the 21 November eruptive event, which we will summarize first. A detailed analysis of our data is presented afterwards.

## Results

**Figure 1:**
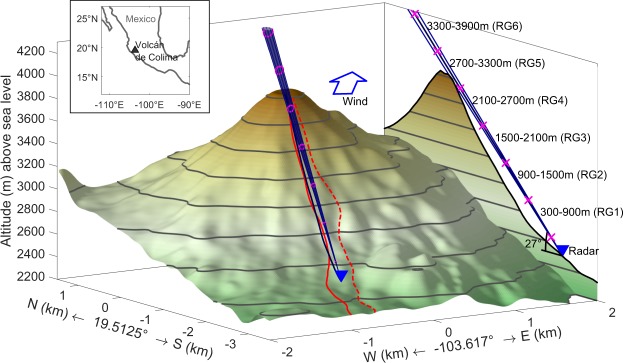
Topography and setup of the Doppler radar (blue triangle) at Volcán de Colima, Mexico. Topography from digital elevation model^39^ with radar beam (grey cone) shown to scale. Blue crosses separate distance intervals for which the range is given. Cross section profile (along black line, deviates 14.24° from N) projected on the right. Range gate length is 600 m, the radar beam centre is at about 100 m height above the dome and has a diameter of 77 m at that distance. The map is centred at the crater at 19.5125°N and −103.617°E. Altitude is colour coded and the same for topography and profile. 200 m contours are shown. Red lines mark possible pathways of the dense ground hugging component of PDCs down the San Antonio (solid) and Montegrande (dotted) ravines. Paths were calculated by following the steepest descent along the topography (see Supplementary Fig. S1, for more details). Note that the pathway of the PDC highly depends on its starting point. PDCs down the dotted line will not be recorded by the radar because of the SSW wind direction on that day. Wind is indicated by the blue arrow and blows toward the NNE (into the figure, see Supplementary Fig. S4).

The Doppler radar was set up roughly 2,700 m slant distance from the crater rim (i.e. approximately 3000 m slant distance from the vents) on the south flank (see Fig. 1, blue triangle), on a ridge in-between the ravines where the PDCs descended (solid and dashed red line, respectively, Fig. 1). The Doppler radar was installed to monitor the volcanic activity by measuring the velocity and echo power (a proxy for the amount of tephra^33^) within the radar beam (cone above the topography in Fig. 1). The radar beam is split into six distance intervals (i.e., range gates). Each of the range gates is a conical volume of 600 m length and an increasing radius with distance to the radar (opening angle of approximately 1.5°). Range gate 5, for example, is centred above the crater and has a mean diameter of 80 m. In each range gate, the velocity component along the radar beam of all particles is measured together with the cumulative reflected power for each velocity. Because the reflected power depends on the concentration and size of particles (for details see ref.^33^) it is only a zero order measure for mass moving inside the nearly cylindrical volume covered by each range gate. For each of these “volumes” the Doppler radar provides information on the velocities of all particles moving inside this volume, i.e. a velocity spectrum. The amplitude (i.e. reflected power) for each measured velocity is a measure of the number of particles (not necessarily of the same size) moving at that speed. We use the term “particles” here to avoid any implicit hints on sizes. Depending on particle concentration, our radar, operating at a wavelength of 1.25 cm, is able to “see” particles with radii larger than a millimetre.

**Figure 2:**
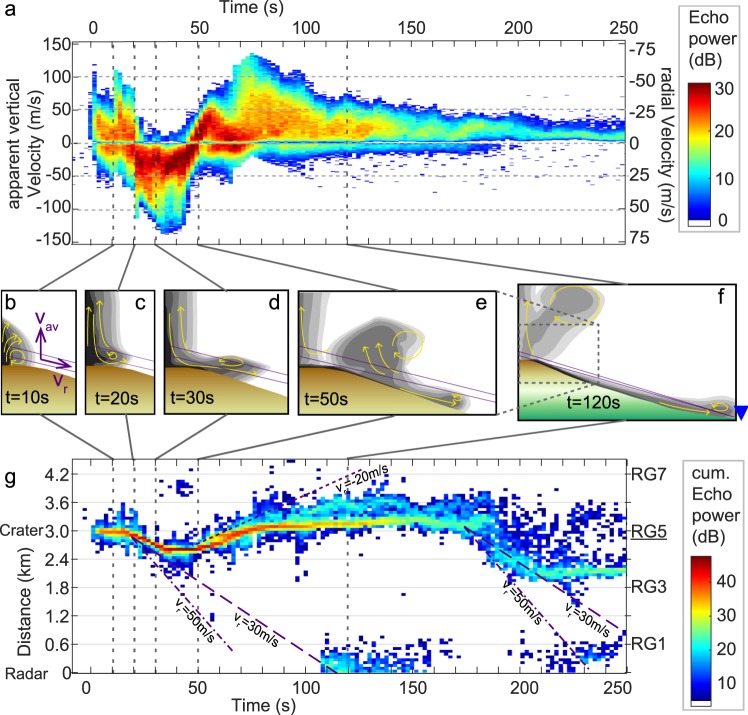
(**a**) Doppler radar data displayed as a velocigram. Each pixel shows the approximate amount of material (echo power in dB, colour coded) moving at a certain velocity (y-axis) and in time (x-axis). The echo power is in dB relative to the noise level. The left y-axis shows apparent vertical velocity (see methods section) and the right y-axis shows measured radial velocity (+ and − indicate toward and away from the radar, resp.). Time is in seconds and zero time corresponds to the beginning of the event at 21 Nov. 2014 at 18:24:24 UTC. Shown is the range gate above the dome, centred at 3,000 m slant distance from the radar (see Supplementary Fig. S2 for the complete data set with all range gates). (**b**–**f**) Schematic drawings of the inferred dynamics within the eruption cloud and PDC at specific times in a cross-section through dome and radar beam (purple lines). Schematic drawings are inspired by a suite of numerical modelling results^40^. Grey scale indicates concentration of particles, yellow arrows show main flow directions. (**b**) Jet-like ejection of particles. The purple arrows point into positive apparent vertical (*v*_*av*_) and radial (*v*_*r*_) velocity, respectively. (**c**) Initiation of collapse as entrainment is not sufficient to reduce concentration in cloud centre. (**d**) Collapse develops into ground hugging dense underflow with turbulently convecting head. (**e**) PDC head and dense underflow travel down the flank below the radar beam. Entrainment in dilute upper part initiates a buoyantly rising thermal, which is re-entrained into the main eruption plume. (**f**) The buoyant thermal merges with the main plume. PDC re-enters the radar beam near the radar where the beam approaches the ground. Note that this schematic drawing shows an extended view, compared to (**b**–**e**). (**g**) Slant distance of the apparent centre of mass (see the methods section and Supplementary Figs S2 and S3) from the radar as a function of time. The colour is a proxy for the amount of material detected at each sub-range gate distance interval. Purple lines in (**g**) indicate average velocities of the PDC moving toward the radar (30 m/s, dashed, and 50 m/s, dash-dotted) and away from the radar (20 m/s, dotted).

The radar data are presented in an overview plot, a so-called velocigram (Fig. 2a), showing the temporal evolution of apparent vertical velocity and echo power in the distance interval covering the summit. Note that we use the term apparent vertical velocity, because the Doppler radar measures the component of a particle’s velocity along the radar beam, termed radial velocity. Under the assumption of vertical motion, and using the known inclination *α* of the radar beam, these radial velocities *v*_*r*_ can be converted to apparent vertical velocities *v*_*av*_ by the trigonometric formula: *v*_*r*_ = *v*_*av*_/sin(*α*). Here, the Doppler radar beam was inclined upward (*α* = −27°), thus radial and apparent vertical velocities are approximately related by a factor of −2. The echo power is the reflected power converted to dB and depends on the amount and size of particles in the volume covered by each range gate (see Fig. 1). In a velocigram of one range gate each pixel thus carries information on how much material (colour) is moving at a specific apparent vertical velocity (vertical axis) at a specific point in time (horizontal axis). Because the particle motion is mainly influenced by gravity and, depending on its size, also by air friction, the temporal evolution of apparent vertical velocities can give information on the size of particles^34^ and atmospheric dynamics^35^.

In the velocigram, the explosive event can be clearly identified as a sudden appearance of fast particles in range gate 5 at 100 m above the vent (Fig. 2a, t = 0 s). Decreasing velocities after the initial peak mark a deceleration phase, which is due to gravity and friction with air^36^. A subsequent pulse at t = 10 s can be identified accordingly (Fig. 2a,b, t = 10 s).

In a typical Vulcanian event at Volcán de Colima, the buoyant rise of an eruption cloud evolves after one or more explosive pulses. A buoyant convecting cloud can be identified in a velocigram as a band of velocities (see Fig. 2a t > 110 s) with similar echo power that extends over tens of seconds. The mean value of this velocity band is the velocity at which the buoyant cloud rises (through the radar beam). For the event reported here, buoyant convection begins at roughly 80 s into the event. The apparently decreasing maximum velocity indicates that the convecting cloud leaves the radar beam and propagates upwards.

For this particular event, however, the explosions at the beginning (Fig. 2a, t = 0 s and t = 10 s) and the typical buoyant convection at the end (Fig. 2a, t > 110 s) are separated by a phase of entirely negative (downward) velocities (Fig. 2a, t = 23–45 s), followed by a phase of almost constant upward acceleration (Fig. 2a, t = 45–55 s). Such dynamics have not been observed at a volcano with radar before. There are three scenarios that can explain the phase of purely negative velocities indicating falling particles: (i) particles ejected at a vent to the left or right of the radar beam fall back to the ground through the beam; (ii) the concentration of falling particles is so high that they create a “curtain”, behind which the rising particles become invisible, due to attenuation of the radar beam; and/or (iii) a PDC has formed that moves toward the radar (Fig. 2d), in which case radial rather than vertical velocities have to be used for interpretation. With a novel technique (see Methods section) to analyse our data, we are able to distinguish between these scenarios and confirm that the latter is the cause of the negative velocities. However, attenuation of the radar beam most likely occurs as well, and the activity above the crater becomes temporally invisible between t = 23–45 s.

**Figure 3:**
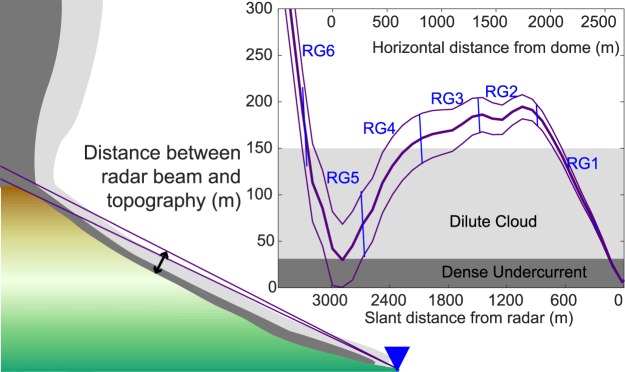
Height of the radar beam above the topography as a function of distance from the vent. The thick purple line follows the centre of the radar beam, thin purple lines indicate the width of the beam (opening angle of 1.5°). Blue lines mark the range gate borders. Assuming, the PDC head to be 150 m high (light grey area), the PDC head can only be seen in RG5, RG4 and RG1, whereas it is invisible to the radar in RG3 and RG2. Assuming that the dense undercurrent (dark grey) has a thickness of 20% of the PDC height^23^, it would only be visible to the radar directly at the crater rim in RG5. The schematic drawing in the lower left is for clarification of the geometry and similar to Fig. 2f.

We use an FMCW-Doppler radar and therefore it is possible (see Methods section) to retrieve the position of the apparent centre of mass at sub-range gate distance resolution (see Fig. 2g). Any PDC following the topography with motion towards the radar will show up successively in different range gates in the data. The event starts at the distance of the dome (approximately 3,000 m slant distance). Most likely the second pulse (Fig. 2, t = 10 s) also emanates from this location. Afterwards, the apparent centre of mass moves toward the radar (see Fig. 2g), its time corresponding to the column collapse (Fig. 2c). The flow front, i.e. the maximum extent of the centre of mass, apparently rests at 2,200 m for a couple of seconds before it moves back toward the crater region. This apparent stagnation of the flow front at 2,200 m (dark blue patches in Fig. 2g) can be explained by the topography dipping below the radar beam (see Fig. 3) so that the dense undercurrent, as well as the dilute cloud of the PDC, leave the radar beam at its base. Note that the radar beam re-approaches the ground at distances closer than 600 m (Fig. 3) and thus parts of the PDC may re-enter the radar beam. Indeed, a second centre of mass at less than 300 m distance (see Fig. 2g,f beginning at 110 s) indicates that the PDC at least travelled this far. Which part of the PDC (the PDC head, the dilute cloud or the dense undercurrent) reached the radar cannot be inferred from the radar data. From Fig. 2g we can see that the apparent centre of mass moved from the crater to the radar location at an average speed of 30 m/s (dashed line). The flow front and thus the fastest particles may have moved initially at 50 m/s (dash-dotted line). Both velocities inferred from the apparent center of mass movement are consistent with the velocigram data (Fig. 2a), where the maximum (radial) velocity toward the radar is >50 m/s and the bulk of material moves at 30 m/s and slower.

**Figure 4:**
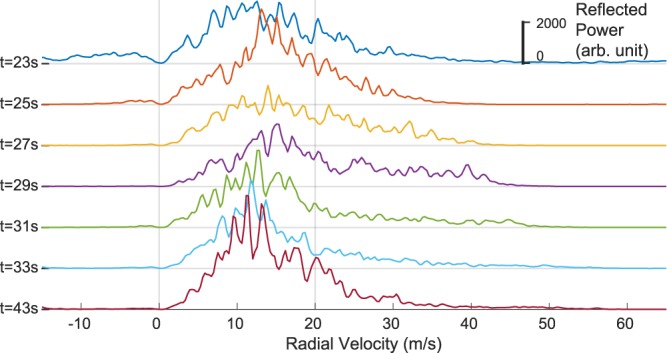
Velocity spectra of range gate 5 at seven different times during the PDC descent. Each velocity spectrum is a “vertical slice” through the velocigram. The vertical scale is the same for all spectra. Reflected power is in arbitrary units (instead of echo power in dB). The time for each spectrum is given on the left and equals the times given in Fig. 2.

Because of the measurement geometry, the radar can see the dense undercurrent of the first PDC only at the crater rim, where the radar beam is near the ground (Fig. 3). Closer to the radar a dense undercurrent following the topography will dip below the radar beam. In Fig. 4 the velocity spectra of the first 12 s of the PDC are shown. At the beginning of the PDC (t = 23 s, Fig. 4) the velocity spectrum shows a wide range of velocities with an equally wide distribution of reflected power (no prominent maximum). The wide velocity spectrum is indicative of convection, with its mean velocity representing the effective velocity of the vortex. This wide spectrum shows the eruption column collapse, when it first hits the ground and moves over the crater rim.

Two seconds later, the shape of the spectrum becomes more peaked (t = 25 s, Fig. 4), which indicates that the main part of the flow moves at 15 m/s toward the radar. The fastest particles inside the radar beam move at 40 m/s. The head vortex of the PDC has left the radar beam and is followed by a less turbulent, more narrowed flow (main body). Another two seconds later (t ≥ 27 s) the velocity spectrum becomes split into two parts: The velocities below 20 m/s have a maximum at 15 m/s (decreasing to 11 m/s in later spectra), whereas velocities above 20 m/s have a linearly decreasing spectrum. The maximum velocity constantly increases from one spectrum to the next up to 60 m/s (t = 43 s). The cumulative reflected power for the fast particles stays approximately constant, whereas the cumulative reflected power for the slow particles increases from 50 to 85% (of the total sum).

The signal between 45–55 s after the onset of the event (Fig. 2a) shows a linear increase in velocity for the bulk of the material, meaning that the acceleration acting on each particle is similar and almost constant. This velocity reversal indicates that the bulk of the (initially falling) material, at least the part inside the radar beam, has become buoyant and experiences constant upward acceleration until it moves upward again (Fig. 2e). This phase of velocity reversal (Fig. 2e,a, t ≈ 50 s) corresponds to the movement of the apparent centre of mass from the flank toward the vent (see Fig. 2g) at an average speed of 20 m/s (dotted lines). This is faster than atmospheric wind speed at this time of day (10 m/s from south-south-west, see Supplementary Fig. S4), which indicates that this phase of velocity reversal is dominated by buoyancy. Our interpretation is that the upper part of the PDC entrained enough ambient air to become buoyant and decouples from the basal flow that continues down the flank below the radar beam (Fig. 2e).

During this event several PDCs descended down the south, south-west and west flanks^29^. We have several indications in our Doppler radar data for at least one second PDC. The buoyant cloud that develops above the PDC would have been transported by atmospheric winds. The prevailing wind direction during this time was from the SSW at 10 m/s (sub-parallel to radar beam, see Supplementary Figs S4 and S1), which means that a buoyant cloud would have been blown towards the NNE. A PDC that follows a path west of the radar beam, can possibly be seen indirectly by its associated buoyant cloud that is blown through the radar beam high above the ground. Beginning at t = 180 s the apparent centre of mass (Fig. 2g) moves toward the radar again and propagates at 30–50 m/s. The apparent centre of mass reaches a distance of 2,000 m (from the radar), which corresponds to the highest beam elevation above the topography (see Fig. 3). The propagation velocity is the same as for the first PDC, but the amount of material inside the radar beam appears to be much less (light blue colour in Fig. 2g). Unlike the first PDC, the velocity spectra show a convection pattern with predominantly upward velocities (see Supplementary Fig. S2, t ≥ 190 s). Thus individual particles move upward (5 to 10 m/s radially away from the radar), but the distance at which particles move through the radar beam shifts toward the radar. This means that the PDC follows a path outside, yet near the radar beam, so that the PDC itself cannot be seen. However, any buoyantly rising material from the PDC rose through the beam. The recorded velocities thus belong to the buoyant cloud above the approaching PDC.

To summarize, the eruptive event started with two successive sub-vertical Vulcanian explosions, separated by 10 s with maximum ejection velocities of >100 m/s (vertical). Immediately after the second explosion the eruption column began to collapse and a PDC emerged from the crater rim descending the south flank (20–45 s since start of eruption, initial velocity of 30–50 m/s). The PDC travelled down the S flank with an average velocity of 30 m/s and finally reached the radar at 110 s. Simultaneously, entrainment of ambient air into the head and dilute upper part of the PDC^23^ led to a density decrease and development of a thermal^11^ moving toward the crater rim (45–80 s, velocity of 20 m/s) and eventually merging to ascend with the remainder of the eruption column (70–100 s). At the crater, buoyant convection successively transported material to greater heights until at 180 s the activity shifted again toward the radar. This indicates a second PDC heading towards the SW.

## Discussion

PDCs can have several causes, one of them being the collapse of the eruption column. Numerical modelling of a collapsing column shows that in this case a momentum driven fountain develops^13,14^. Such motion appears in a velocigram as linearly decaying velocities (i.e. constant gravitational acceleration), which can be seen in Fig. 2a, t ≈ 20 s. Other causes for PDCs, such as gravitational breakup of a lava dome or a directed explosion would experience different starting velocities and acceleration scenarios, which would appear in a velocigram with their respective typical signature^37^. As neither a constant acceleration of initially resting material (gravitational break-up), nor a sudden appearance of apparently falling material without it being ejected upward in advance can be seen, we favour the eruption column collapse-scenario for the first PDC. For the second PDC, there is no indication of a vertical explosion with subsequent column collapse. Therefore we suggest that the second PDC is generated by gravitational break-off of dome material descending the flank outside the radar beam after the first explosion destabilized the dome^3^.

From large scale experiments it is known that the highest velocity inside a PDC is near the ground, slightly above the boundary between dense undercurrent and dilute cloud^19,23,38^. Both parts of the PDC (dense undercurrent and dilute cloud) move at comparable velocities. The particle volume concentration, however, is two to three orders of magnitude larger in the dense undercurrent^23^ and so the reflected power is orders of magnitude larger. Therefore, when looking into an experimental PDC, the radar would mainly record the dynamics of the dense undercurrent, the contribution of the dilute cloud being negligible. The velocity spectra that we measured at Volcán de Colima, however, show two distinctly different parts (see Fig. 4): The main body, moving slower than 20 m/s with a gradual increase in concentration, and a less dense fast part (>20 m/s) with approximately constant concentration.

The main difference between experiments and numerical eruption column models to natural PDCs is the particle size distribution. Scaled experiments use particle diameters <1 cm, while numerical eruption column models often assume particle sizes of less than a few millimetres, due to approximations in the model equations^14^. In deposits of natural PDCs, however, all particle sizes up to several metres in diameter can be found^32^. In fact, particles >1 cm are most abundant and their influence on the PDC dynamics must be taken into account. Numerical models for granular flow or dry avalanches^15,16^ make no assumptions on particle sizes. Instead the dense undercurrent is modelled as a fluidized mixture of particles (all sizes) and gas whose flow is governed by internal friction. These models, however, cannot describe the dilute cloud atop the dense undercurrent.

With this Doppler radar we measure the velocity of large particles (>1 mm) as they reflect more of the electromagnetic wave than small particles (<1 mm) assuming the same concentration. We interpret the two parts of the velocity spectra as dense undercurrent of the PDC (<20 m/s) overflowing the crater rim and being fed by the fountain-like eruption column that is invisible to the radar due to complete attenuation of the radar beam by the dense undercurrent. The dense undercurrent dips below the radar beam at around 2600 m distance from the radar (100 m below the crater rim). The fast particles (>20 m/s) were accelerated within the collapsing column by gravity and decouple from the dense undercurrent. These fast particles are visible in the radar beam up to a distance of 2200 m from the radar, by which time the dense undercurrent has already left the beam. The fastest particles (larger than 1 mm) thus move in suspension with the dilute cloud to greater heights above the topography than has been previously seen in experiments. The timing of the first arrival of particles near the radar, where they still have a maximum velocity of 15 m/s, indicates that the cloud front at least travelled with a velocity of 30 m/s and a significant proportion was comprised of mm- to cm-sized particles.

The separation of a dense undercurrent and dilute upper part have been investigated experimentally^19,23^. The development of thermals from the dilute upper part of the density current and the subsequent merging with the eruption column have already been simulated numerically^13,14^. The abundance of large particles in the PDC has been reported from deposit-studies, but not included into numerical models because of the lack of an appropriate parametrisation of the interaction between gas and particles larger than a few millimetres^14^. There exists one set of experiments^23^ that investigates the feedback of particles up to 8 mm diameter on the PDC flow structure and dynamic pressure during flow. They show that “larger” particles should be taken into account for a correct estimation of dynamic pressure and thus hazard mitigation. We here present for the first time a data set of internal particle velocities during column collapse, and beginning of a PDC that can now be used, together with a realistic particle size distribution (e.g. inferred from deposits) as well as the topography as the input to numerical models for the simulation of PDCs. Because we know the approximate path of the PDC while approaching the radar, as well as the run-out distance of at least 3 km, we can evaluate whether state-of-the-art numerical models (which are currently used for hazard assessment studies) are able to replicate this particular PDC. Such a comparison between data and models has given valuable insights into the dynamics of snow avalanches^25^ and could now be done for volcanic PDCs.

## Methods

### The FMCW-Doppler radar

Radar makes use of reflection and scattering of electromagnetic waves by objects. A radar typically transmits a signal (pulsed or continuous wave, CW) and records the reflected incoming signals as well as the round-trip travel time, which is directly proportional to the distance of the reflector. The amplitude of the incoming signal is related to the size and shape of the target object (ref.^33^ and references therein). For distributed targets, like rain or volcanic tephra, the amplitude relates to the target’s size and concentration. However, it is not possible to record individual round-trip travel times for distributed targets, and therefore the incoming signal is divided into time intervals, which directly translate into distance intervals, the so called range gates. To measure round-trip travel times with a CW radar a modulation of the transmitted wave is required (a pulse is an extreme case of amplitude modulation). The FMCW radar uses frequency modulation (FM, here a decreasing sawtooth shape). The travel time can now be determined from the frequency difference of simultaneously received and transmitted signal. The larger this difference, the longer the travel time. In practice, both the incoming and transmitted signals are mixed and low-pass filtered, such that the mixed signal only contains the difference frequencies (of multiple targets). This mixed signal is then analysed using a Fourier transform; the resulting power spectrum is a histogram of material at various distances, irrespective of their velocity.

A radar has Doppler capability, meaning that in addition to amplitude and distance, it also provides velocity information. When targets move within the radar beam, the distance between radar and target (i.e. the round-trip travel time or frequency difference) changes between two measurements. These changes are recorded as a phase-change of the continuously received incoming signal. This phase-change is directly proportional to the targets radial (along-beam) velocity and is obtained via a Fourier transform of the distance measurement. The result is a histogram of target velocities for each range gate (see Supplementary Fig. S3).

### Retrieval of the centre of mass

The main difference between pulsed- and FMCW-Doppler radars lies in the interpretation of the amplitude in neighbouring range gates. A pulsed radar uses a time interval to assign parts of the signal to range gates, so that targets exclusively appear in their correct range gate^35^. This is different for FMCW-Doppler radar, as the signal is analysed using a Fourier transform, which suffers from spectral leakage. In a discrete Fourier transform, spectral leakage “smears” the amplitude that belongs to one specific frequency across neighbouring frequencies. This means, that each target will appear in its correct range gate, but also in the neighbouring range gates, however, with a much smaller amplitude. In the internal processing of our FMCW-radar, leakage affects roughly the three nearest neighbours on both sides of the true frequency. Spectral leakage occurs for both, the range and the velocity Fourier transform, but can be neglected for the calculation of velocities, because we chose a high velocity resolution of 0.28 m/s. For the less well resolved range gates (600 m), the spectral leakage effect is significant, but also contains additional information.

A PDC, or a dense cloud of particles, approaching the Doppler radar will cross one range gate after the other. Thus the amplitude (or the reflected power) moves across the range gates. Using a pulsed radar, where every particle appears exclusively in its associated range bin, the PDC will appear to approach the radar in steps (with step length equal to range gate length). In the FMCW-radar, however, the range is determined via Fourier transform that suffers from spectral leakage. To reduce this leakage effect, a Gaussian window is applied before the Fourier transform. This Gaussian window forces the leaked amplitudes to a well-defined Gaussian curve shape, with its maximum at the true frequency (which might lie between the Fourier transform’s frequency bins).

Fitting the well-defined Gaussian curve to the leaked amplitudes (using the Levenberg-Marquardt algorithm) gives the amplitude and position of the maximum of the Gaussian curve, which corresponds to the true distance of the reflector. This can be done for each velocity sample separately (see Supplementary Fig. S3, for a graphical explanation of the method). For distributed targets (like rain drops or volcanic tephra) one velocity sample contains information of how much material (reflected power) moves with the respective velocity in the radar beam at various distances (range gates). The algorithm returns the apparent centre of mass for each respective velocity sample in sub-range gate resolution. Because the analysis is done for each velocity sample independently, the algorithm can resolve different parts of the particle cloud that move at different distances and velocities.

For dense clouds, however, attenuation of the radar beam will limit the distance the radar beam penetrates into the cloud. Thus the reflections all correspond to this limited distance and the derived centre of mass also belongs to this limited part of the cloud. Therefore we named our parameter “apparent” centre of mass, because it is the centre of the attenuated path length, which, depending on particle concentration, is shorter than the length of the radar beam crossing the particle cloud. Thus the true centre of mass can be further away from the radar than the apparent centre of mass.

The propagation velocity of the centre of mass may or may not be related to the velocity of individual particles and/or the front of the particle cloud. For an individual batch of particles, the propagation velocity of the centre of mass will equal the mean velocity of particles, i.e. the true propagation velocity of the batch (as long as it moves along the radar beam). For a continuously fed flow toward the radar, and neglecting attenuation of the radar beam, the velocity of the flow front will be twice the propagation velocity of the centre of mass. Considering attenuation, the apparent centre of mass will move with a velocity between those two end-member case scenarios.

The range gate resolution of FMCW-Doppler radars can thus be highly increased by fitting a Gaussian curve to the data. Using this trick, the sub-range resolution outperforms pulsed-Doppler radars because FMCW-Doppler radars do not suffer from the inability to correctly identify particles that move faster than some maximum unambiguous velocity.

## Supplementary information

Supplementary Figures

## Data Availability

The data is available upon request.
