# Supplementary material for: First in-situ observation of a moving natural pyroclastic density current using Doppler radar
Source: Sci Rep. 2019 May 14;9:7386. doi: 10.1038/s41598-019-43620-w (PMC6517430; doi:10.1038/s41598-019-43620-w)
Supplement: Supplementary file 1 — Supplementary Figures [file 41598_2019_43620_MOESM1_ESM.pdf]

Supplementary Information to ”First in-situ observation of a moving natural pyroclastic density current using Doppler radar” by L. Scharff, M. Hort, and N.R. Varley

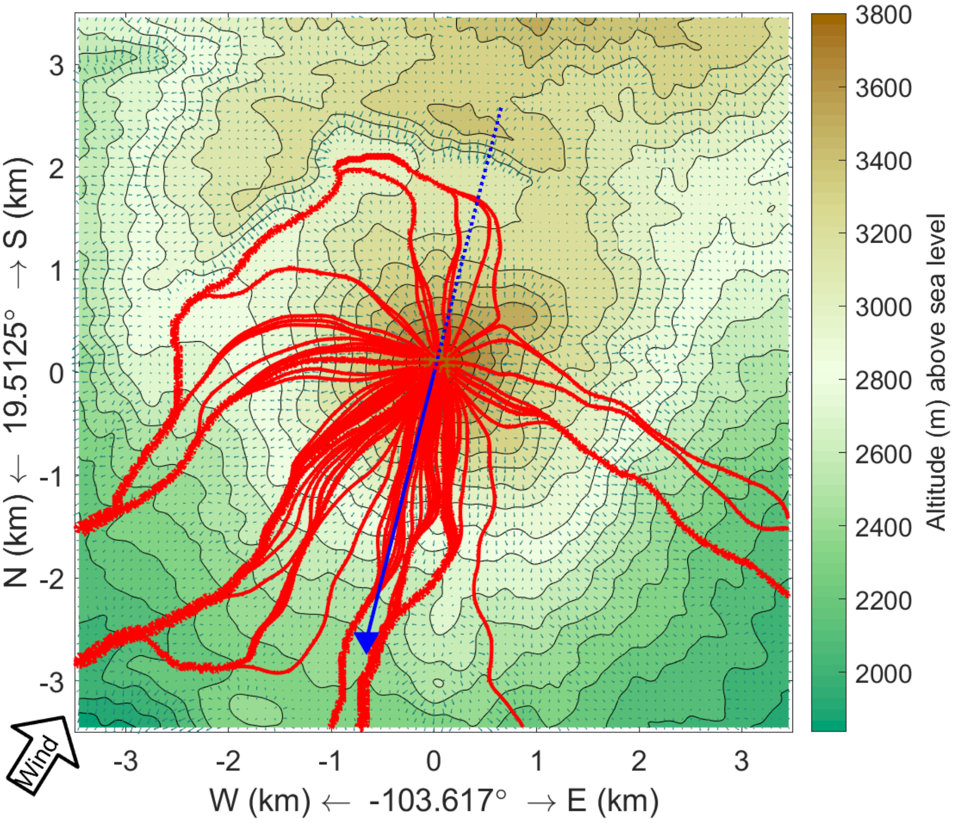

Supplementary Information, Figure S1: Possible pathways of pyroclastic density currents (PDC) and lahars originating at different points in and around the crater. Contour-level spacing is 100 m. We use the 3 arc second digital elevation model (see Fig. 1) to calculate local gradients. Using a grid of 9 by 9 points on the mountain top as starting points (grid spacing of 50 m) we calculate and plot the paths of locally steepest descent for each of the 81 starting points. When two or more lines merge in valleys, the line thickness increases. Several preferred drainages to the SW, S and SE can be identified, which correspond to specific ravines known for PDC runouts. Note that inertia and overtopping are not included in this first order approximation and that pyroclastic density currents may also travel E and NE once they overtop Volcancito dome (plateau NE of the crater). The radar (blue triangle) was located between the San Antonio and Montegrande ravines (W and E from radar, respectively). The radar beam (blue line) is plotted for reference. Note that it extends beyond the crater (dotted line), rising into the sky. When pyroclastic density currents travel along the radar beam or wind-blown particles cross it, they are visible to the radar. The black arrow in the lower left corner indicates the prevailing wind direction on 21 November 2014 (18-21h UTC) during the pyroclastic flows analysed in the main article. Information on wind direction is taken from reanalysis data (see Supplementary Figure 4). Despite the simplicity of the analysis, this map compares well to hazard maps generated for PDCs at Volcán de Colima, published in the Atlas de Riesgos Estatal (http://www.atlascolima.org/geoweb/).

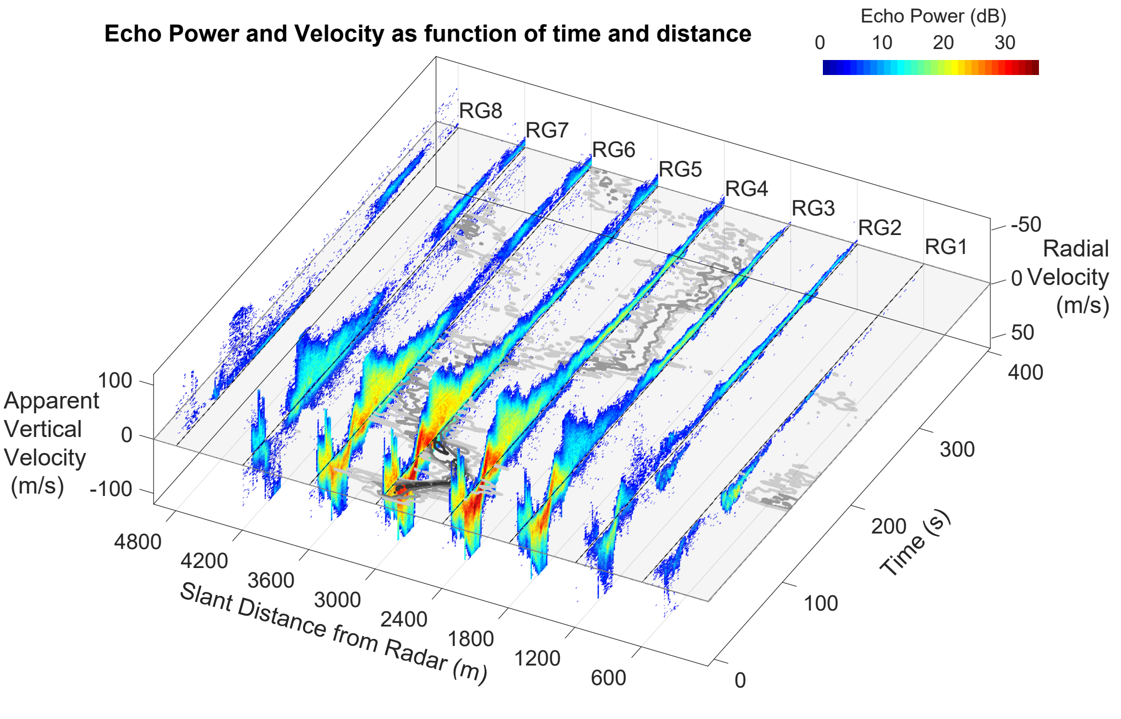

Supplementary Information, Figure S2: The complete Doppler radar data set of the first 400 s of the event on 21 Nov. 2014, 18:24:24 UTC plotted as velocigrams of all range gates as function of distance from the radar. The velocigram at 3000 m distance is the same as in Fig. 3a. Data are noise reduced and converted to apparent vertical velocity (left ticks). For comparison radial velocity is given on a second velocity axis (right). Colours correspond to echo power in dB (above noise level). Echo power below the transparent plane at zero velocity belongs to falling particles or particles approaching the radar. Echo power above the transparent plane belongs to particles moving upward or departing from the radar. The grey scale contours on the transparent plane show the cumulative echo power as a function of distance (same as Fig. 2g). Contour lines are plotted for 10, 20, 30, and 40 dB from light grey to black. The sub-range distance resolution is obtained from the relative amplitudes of neighbouring range gates (see Methods Section and Supplementary Figure 3). Note that due to internal processing reasons there exist no data for positive velocities at 600 m (RG1) and negative velocities at 4800 m (RG8).

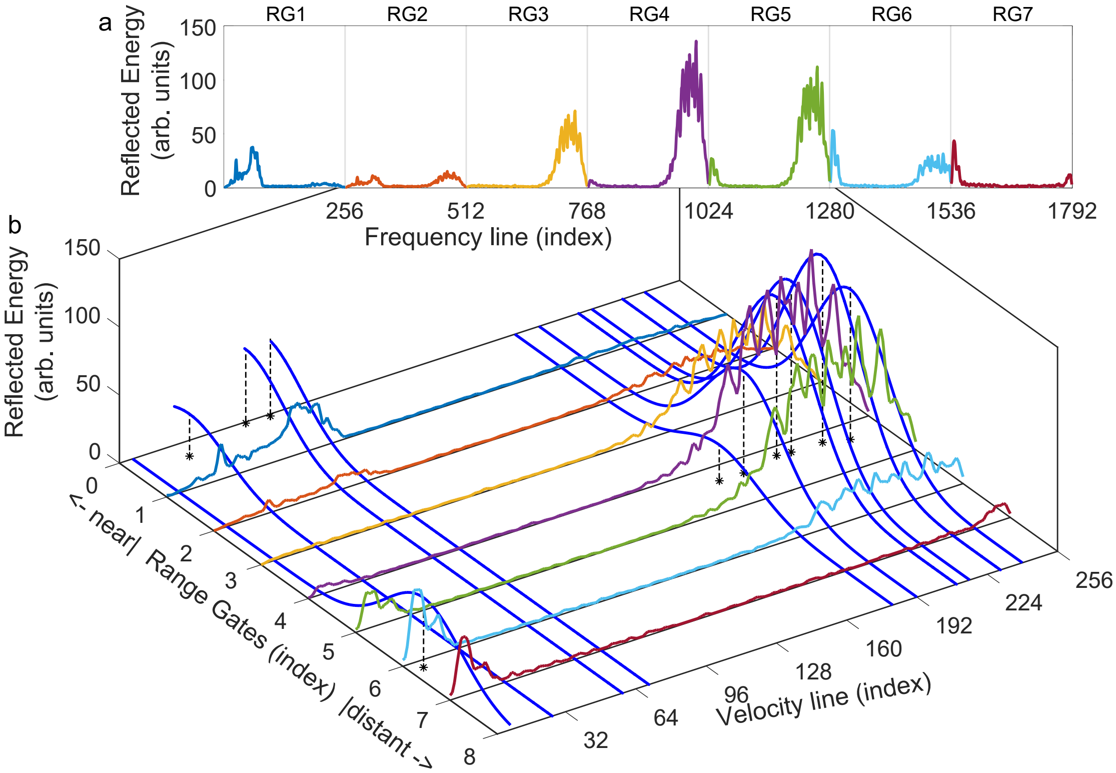

Supplementary Information, Figure S3: Graphical explanation of the retrieval of sub-range gate resolution. a) One velocity spectrum measured at a specific time (here 117 s into the eruption). Each colour represents the velocity spectrum within one range gate. Each of these velocity spectra can be viewed as a histogram of velocities (bin width of dv) at the respective distance. The frequency difference between outgoing and incoming signal corresponds to the sum of travel time and Doppler effect-induced frequency shift. Hence, each measured value has an associated frequency line index i_f that represents both range gate number i_{rg} and velocity bin i_v: $i_f = (i_{rg} - 1) * 256 + i_v$. The radial velocity v_{rad} is then given by $v_{rad} = (128 - i_v) * dv$. b) Resorting of the velocity spectrum separates range gate and velocity line indices. For each velocity line, we fit one Gaussian curve (dark blue lines, only the curves with the largest amplitudes are shown), with its maximum position corresponding to the true distance of the material with this specific velocity (stars projected on bottom plane). The amplitude of the Gaussian curve corresponds to the echo power of material at that specific distance and velocity. Because the fit is done for each individual velocity line, we can identify moving particles at different distances as long as they move at different velocities (i.e. line 8 in RG6, line 64 near RG0, lines 192–256 between RG4 and RG5).

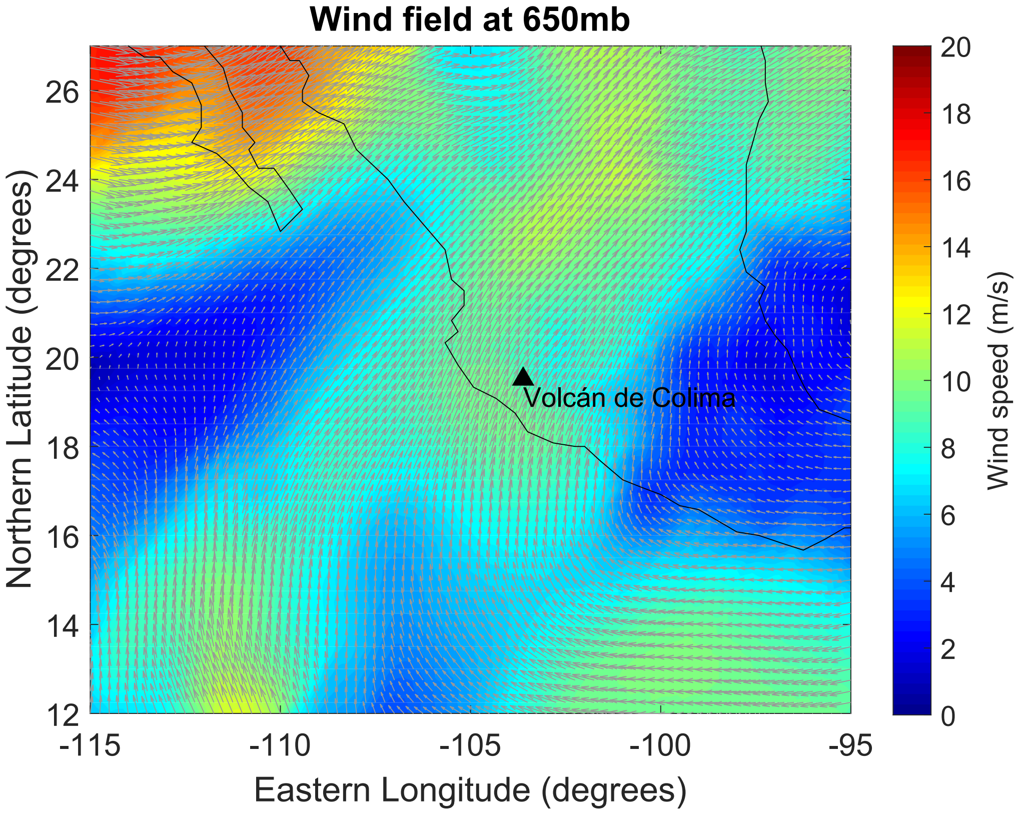

Supplementary Information, Figure S4: Wind speed (colours) and direction (arrows) at 650 mb pressure level, which near the volcano corresponds to a height of 3775.8 m above sea level (i.e. below the crater rim at 3850 m) on November 21st, 2014, 18:00-21:00 UTC. Grid resolution is 0.3x0.3° (grey arrow at each grid point). Black line is the Mexican coast line, black triangle marks the location of Volcán de Colima, Colima, Mexico. The wind speed at the time of the pyroclastic density current was 10 m/s and blowing from south-southwest direction. Data from NCEP North American Regional Reanalysis: NARR, a high resolution combined model and assimilated dataset. NCEP Reanalysis data provided by the NOAA/OAR/ESRL PSD, Boulder, Colorado, USA, from their Web site at https://www.esrl.noaa.gov/psd/
